# Large Language Models’ Accuracy in Emulating Human Experts’ Evaluation of Public Sentiments about Heated Tobacco Products on Social Media: Evaluation Study

**DOI:** 10.2196/63631

**Published:** 2025-03-04

**Authors:** Kwanho Kim, Soojong Kim

**Affiliations:** 1 Department of Media College of Politics and Economics Kyung Hee University Seoul Republic of Korea; 2 Department of Communication University of California Davis Davis, CA United States

**Keywords:** heated tobacco products, artificial intelligence, large language models, social media, sentiment analysis, ChatGPT, generative pre-trained transformer, GPT, LLM, NLP, natural language processing, machine learning, language model, sentiment, evaluation, social media, tobacco, alternative, prevention, nicotine, OpenAI

## Abstract

**Background:**

Sentiment analysis of alternative tobacco products discussed on social media is crucial in tobacco control research. Large language models (LLMs) are artificial intelligence models that were trained on extensive text data to emulate the linguistic patterns of humans. LLMs may hold the potential to streamline the time-consuming and labor-intensive process of human sentiment analysis.

**Objective:**

This study aimed to examine the accuracy of LLMs in replicating human sentiment evaluation of social media messages relevant to heated tobacco products (HTPs).

**Methods:**

GPT-3.5 and GPT-4 Turbo (OpenAI) were used to classify 500 Facebook (Meta Platforms) and 500 Twitter (subsequently rebranded X) messages. Each set consisted of 200 human-labeled anti-HTPs, 200 pro-HTPs, and 100 neutral messages. The models evaluated each message up to 20 times to generate multiple response instances reporting its classification decisions. The majority of the labels from these responses were assigned as a model’s decision for the message. The models’ classification decisions were then compared with those of human evaluators.

**Results:**

GPT-3.5 accurately replicated human sentiment evaluation in 61.2% of Facebook messages and 57% of Twitter messages. GPT-4 Turbo demonstrated higher accuracies overall, with 81.7% for Facebook messages and 77% for Twitter messages. GPT-4 Turbo’s accuracy with 3 response instances reached 99% of the accuracy achieved with 20 response instances. GPT-4 Turbo’s accuracy was higher for human-labeled anti- and pro-HTP messages compared with neutral messages. Most of the GPT-3.5 misclassifications occurred when anti- or pro-HTP messages were incorrectly classified as neutral or irrelevant by the model, whereas GPT-4 Turbo showed improvements across all sentiment categories and reduced misclassifications, especially in incorrectly categorized messages as irrelevant.

**Conclusions:**

LLMs can be used to analyze sentiment in social media messages about HTPs. Results from GPT-4 Turbo suggest that accuracy can reach approximately 80% compared with the results of human experts, even with a small number of labeling decisions generated by the model. A potential risk of using LLMs is the misrepresentation of the overall sentiment due to the differences in accuracy across sentiment categories. Although this issue could be reduced with the newer language model, future efforts should explore the mechanisms underlying the discrepancies and how to address them systematically.

## Introduction

Heated tobacco products (HTPs) are emerging tobacco products that heat processed tobacco leaves, enabling users to breathe nicotine into their lungs [1]. As these products gain global market share at a rapid pace, their potential impacts on tobacco prevention and cessation initiatives are becoming an important topic of public debate [2].

Social media platforms are where a wide range of stakeholders of tobacco regulations distribute their messages, such as policy announcements, product advertisements, and product user feedback [3-5]. Analyses of social media discourses on HTPs provide opportunities to observe and identify the dynamics of these messages, which could affect the public’s perception of these products and relevant regulatory issues [3-7].

Sentiment analysis is a widely adopted method to understand the attitudes of the public toward tobacco-related issues by evaluating social media messages [8-11]. Prior research has specifically focused on positive and negative sentiments due to their possible associations with tobacco cessation and prevention outcomes, such as the use of tobacco products and support for tobacco regulations [11-13].

In past sentiment analyses of large-scale social media content data, human evaluators often examined a subset of the dataset rather than analyzing the entire dataset. The subset was then used as a representative sample to inform the sentiment of the whole dataset [8,9] or as a reference for machine learning classifiers tasked with analyzing the entire dataset [14]. This approach stems from the time-consuming and labor-intensive nature of human sentiment evaluation, which involves recruiting, training, and coordinating multiple evaluators. This complexity arises from the fact that latent coding, including sentiment analysis, requires understanding the underlying meanings and subtleties in the text, which can substantially vary depending on the context and across coders [15].

Large language models (LLMs), such as OpenAI’s Generative Pre-trained Transformer and Google’s Gemini, may be able to alleviate the burdens of human sentiment evaluation. LLMs are artificial intelligence (AI) models that were trained on extensive text data to emulate the linguistic patterns of humans [16,17]. Recent LLMs are known to achieve precision at the level of human decisions on several intellectual tasks [18]. As LLMs become increasingly accessible and available, expectations are growing about the feasibility of using these technologies in public health and social science research [19]. Several examples include analyzing health and medical information [20,21], pretesting the effect of health campaign messages [22], predicting psychological experimental results [23], and simulating sociodemographic groups and their reactions to social issues [24].

We investigate the accuracy of LLMs in analyzing sentiment in social media messages about HTPs. The current research focuses on OpenAI’s GPT, given its high accessibility, availability, and popularity. GPT models are easily accessible through chatbot services such as ChatGPT, Microsoft Copilot, and Apple Intelligence and are estimated to have the largest user base worldwide. For instance, ChatGPT has more than 200 million weekly active users as of August 2024 [25]. These aspects contribute to the attractiveness of GPT as an analytic tool for tobacco researchers, especially those with limited budgets and resources.

This study examines the accuracy of GPT-3.5 and GPT-4 Turbo in emulating human sentiment evaluations of social media messages related to HTPs. GPT-3.5 is a milestone model that powered ChatGPT when the service was launched [26]; GPT-4 Turbo is one of the most recent GPT-4 models as of 2024, with a particular development focus on improvements in processing text prompts [27]. This study conducted direct comparisons of the sentiment evaluations made by human coders and these language models based on social media messages gathered from multiple platforms. This investigation could ultimately contribute to assessing the ability of LLMs in examining how the public views alternative tobacco products.

## Methods

### Data Collection

Messages relevant to HTPs were collected from 2 social media platforms that provide distinct message formats: Facebook (long format) and Twitter (short format). Facebook posts were collected using CrowdTangle (CT)’s keyword search feature. CT was a social media analytic tool provided by Facebook’s parent company, Meta. It allowed researchers to access the historical data of Facebook [28]. Tweets were gathered using Twitter’s application programming interface 2.0, which could access the historical Twitter dataset through the company’s academic research access program [29]. This study focused on messages written in English.

In April 2022, a keyword search was conducted using the following search query: “heat not burn” OR “heat-not-burn” OR “heated tobacco” OR “tobacco heating” OR ((htp OR hnb) AND (smoking OR smoke OR vaping OR vape OR tobacco OR cig OR nicotine)) OR iqos. This query was designed to find tweets and Facebook posts meeting at least one of the following conditions: (1) Containing “heat not burn” in its entirety; (2) containing “heat-not-burn” in its entirety; (3) containing “heated tobacco” in its entirety; (4) containing “tobacco heating” in its entirety; (5) containing at least one of “htp” and “hnb,” only when it also contains one of “smoking,” “smoke,” “vaping,” “vape,” “tobacco,” “cig,” and “nicotine;” and (6) containing “iqos.” This search yielded 16,284 Facebook posts that were published between January 2014 and December 2021 and 60,031 tweets published in the same period.

### Human Evaluation

The procedures for preparing samples for human sentiment evaluations were adapted from sentiment analyses of tobacco-related mass and social media discussions [8-11]. A team of 3 human coders evaluated the sentiment of 1250 Facebook posts and 1200 tweets sampled from the entire pool of keyword-searched social media messages (ie, 16,284 Facebook posts and 60,031 tweets). Those messages were human-labeled as one of the following 5 categories: ANTI (anti-HTP messages), PRO (pro-HTP messages), NEU (neutral messages), MIX (messages containing a mixture of positive and negative attitudes on HTPs), and IR (messages irrelevant to HTPs).

The messages were sampled through a multistep process designed to increase the likelihood of including both potentially negative and positive messages, ensuring their inclusion in the selected messages for human annotation. The details of the sampling and coding procedures are reported in Multimedia Appendix 1.

### GPT Evaluation

From each of the human-evaluated 1250 long-form and 1200 short-form messages, we randomly selected 200 PRO, 200 ANTI, and 100 NEU messages, totaling 1000 messages. All these selected messages for GPT-3.5 and GPT-4 Turbo sentiment classification were relevant to HTPs. A LLM prompt was created for each message, including coding instructions, the message, and the coding scheme. Since this study aims to conduct direct comparisons between the sentiment evaluations of human coders and the language models, the instructions and the coding scheme for the language models were kept consistent with those for human coders, aside from minor formatting adjustments. The instructions included in the prompt directed a language model to categorize a given message based on the coding scheme and to format its response based on formatting rules. The coding scheme, largely identical to the one given to human evaluators, included the definitions and explanations of HTPs and 5 sentiment categories (ANTI, PRO, NEU, MIX, and IR).

LLMs generate a sequence of words by selecting each word based on its preceding words, and the selection is done by sampling a word from a large distribution of possible words [16,17]. Because of the inherent randomness in this sampling process, LLMs may produce different responses to the same prompt. This potential variability can be accounted for by generating multiple responses from an LLM using the same prompt [30]. To be specific, we collected 20 instances of responses for each message, referred to as “response instances.” Each instance was obtained by initiating a new chat with a language model, sending the prompt, and saving the response from the model.

A language model’s decision for each message was determined by randomly selecting *m* instances from a pool of 20 response instances with replacement. Then, the majority within the selected instances was identified. This majority outcome was termed the “machine decision.” We assessed the machine decision by varying the number of response instances (*m*=1, 3, 5, 7, 9, and 11). For example, *m*=5 simulates a scenario where a user generates 5 response instances and identifies the majority among them. *m*=1 corresponds to a “one-shot” determination, where a single instance was generated and considered as the machine decision. In the case of a tie, an extra response instance was randomly selected until the tie was broken.

For each message and each value of *m*, the process of determining a machine decision was iterated 1000 times. After each iteration, a variable that we refer to as “human-machine concurrence” was recorded as 1 if the machine decision aligned with the human evaluation of the message. Otherwise, it was recorded as 0. This variable was then averaged across all iterations, yielding a value referred to as “accuracy.” Thus, the accuracy in this study indicates how accurately the language models classify the sentiment of a message based on the *m* number of responses. Alternatively, the accuracy can be interpreted as the proportion of messages classified by the language models that match the human sentiment evaluation of the same messages. For instance, if the accuracy of a language model is 90% for ANTI message classification, this suggests that nine-tenths of human-labeled ANTI messages are categorized as ANTI by the model. To assess the overall accuracy of the model in evaluating the sentiment of a specific set of messages (eg, Facebook messages classified as ANTI by human evaluators), we calculated the average accuracy across messages in each set, denoted as *K_m_*. For example, *K_11_* for ANTI messages refers to the proportion of human-labeled ANTI messages that were also classified as ANTI by a language model based on 1000 iterations of the majority of randomly selected 11 responses out of the total of 20 responses. Examples of human-labeled ANTI, PRO, and NEU messages, along with the language models’ sentiment classification decisions on the same messages, are provided in Table S16 in Multimedia Appendix 1.

## Results

### GPT-3.5 Evaluation

The average accuracy, based on 20 response instances (*K_20_*), was 0.612 (SE 0.02) for long-form and 0.570 (SE 0.02) for short-form messages. However, the accuracy varied across categories. For messages categorized as ANTI by human evaluators, *K_20_* was 0.755 (SE 0.028) for long-form and 0.696 (SE 0.03) for short-form messages. For messages categorized as PRO by human evaluators, the accuracy was 0.544 (SE 0.033) for long-form and 0.476 (SE 0.033) for short-form messages. The language model’s average accuracy for messages classified as NEU by human evaluators was 0.461 (SE 0.04) for long-form and 0.507 (SE 0.042) for short-form messages. Table 1 presents the accuracy of different sentiment labels with varying *m*.

**Table 1:** Accuracy varying the number of response instances (GPT-3.5).

Platform (format) and *m*^a^			ANTI^b^ (n=200)					PRO (n=200)^c^					NEU (n=100)^d^					All (N=500)		
			*K*_*m*_^e^ (SE)		*K*_*m*_/*K*_*20*_, %		*K*_*m*_ (SE)			*K*_*m*_/*K*_*20*_, %		*K*_*m*_ (SE)			*K*_*m*_/*K*_*20*_, %		*K*_*m*_ (SE)			*K*_*m*_/*K*_*20*_, %
**Facebook (Long format)**																				
	1	0.657 (0.023)		87.1		0.452 (0.025)			83		0.428 (0.024)			92.7		0.529 (0.015)			86.5	
	3	0.715 (0.025)		94.7		0.506 (0.029)			93.1		0.446 (0.030)			96.7		0.578 (0.017)			94.4	
	5	0.734 (0.026)		97.2		0.524 (0.030)			96.2		0.451 (0.033)			97.7		0.593 (0.018)			96.9	
	7	0.740 (0.027)		98		0.530 (0.031)			97.4		0.455 (0.034)			98.6		0.599 (0.018)			97.9	
	9	0.744 (0.027)		98.6		0.535 (0.031)			98.2		0.456 (0.036)			98.7		0.603 (0.019)			98.5	
	11	0.749 (0.027)		99.2		0.537 (0.032)			98.7		0.459 (0.037)			99.5		0.606 (0.019)			99.1	
	20	0.755 (0.028)		—^f^		0.544 (0.033)			—		0.461 (0.040)			—		0.612 (0.020)			—	
**Twitter (Short format)**																				
	1	0.614 (0.025)		88.3		0.411 (0.025)			86.4		0.444 (0.026)			87.6		0.499 (0.016)			87.5	
	3	0.662 (0.027)		95.1		0.449 (0.029)			94.3		0.481 (0.033)			94.8		0.540 (0.018)			94.8	
	5	0.675 (0.028)		97.0		0.459 (0.031)			96.5		0.492 (0.036)			97.1		0.552 (0.019)			96.9	
	7	0.681 (0.029)		97.9		0.465 (0.031)			97.6		0.496 (0.038)			97.9		0.557 (0.019)			97.8	
	9	0.685 (0.029)		98.4		0.469 (0.032)			98.6		0.502 (0.039)			98.9		0.562 (0.019)			98.6	
	11	0.688 (0.029)		99		0.470 (0.032)			98.8		0.502 (0.040)			99		0.564 (0.020)			98.9	
	20	0.696 (0.030)		—		0.476 (0.033)			—		0.507 (0.042)			—		0.570 (0.020)			—	

^a^*m* indicates the number of response instances used for majority determination. When *m* is greater than 1, machine decision is the majority among the response instances. When *m* equals 1, the machine decision is equal to the response instance (1-shot determination).

^b^anti-HTP messages.

^c^pro-HTP messages.

^d^neutral messages.

^e^*K_m_* indicates the average of the machine accuracy of *n* messages when the machine decision for each message was determined based on *m* response instances.

^f^Not applicable.

Most discrepancies arose when the language model classified messages as NEU or IR, whereas human evaluators identified positive or negative sentiments in these messages. For instance, the model misclassified 24.5% (49/200) of human-evaluated long-form ANTI messages. Among these misclassified messages, the language model classified 61.2% (30/49), 26.5% (13/49), and 12.2% (6/49) as NEU, IR, and PRO. Tables S2, S4-S7, and Figures S1 and S2 in Multimedia Appendix 1 provide more detailed comparative descriptions of decisions made by human evaluators and GPT-3.5.

The significance of differences in accuracy between sentiment categories was examined. The results indicated that *K_20_* for human-evaluated ANTI messages was significantly higher than that of human-labeled PRO (*U*=26483.5; *P*<.001) and NEU (*U*=14675.5; *P*<.001) messages in long form. This was also true for human-labeled short-form PRO (*U*=25876.5; *P*<.001) and NEU (*U*=13312.5, *P*<.001) messages. These gaps in accuracy were consistent across all *m* values and formats (see Tables S12 and S13 in Multimedia Appendix 1).

**Figure 1:**
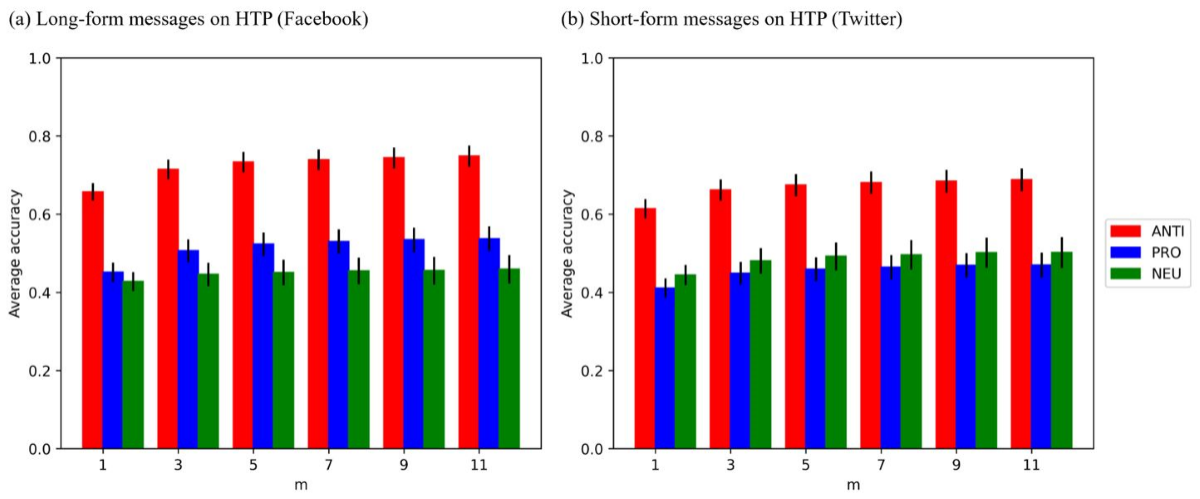
Accuracy across response instances and message formats (GPT-3.5). HTP: heated tobacco product. ANTI: anti-HTP; PRO: pro-HTP; NEU: neutral.

The accuracy improved as *m* increased, as visualized in Figure 1. In Figure 1, error bars represent the mean (SE) of the mean, and “m” refers to the number of response instances. However, even with a few response instances, the accuracy was comparable to the accuracy based on 20 response instances. For example, even the average accuracy of 1-shot determination (*K_1_*) for human-labeled ANTI, PRO, and NEU messages also reached 87.1%, 83%, and 92.7% of *K_20_* for long-form messages and 88.3%, 86.4%, and 87.6% of *K_20_* for short-form messages.

### GPT-4 Turbo Evaluation

GPT-4 Turbo demonstrated higher accuracy than GPT-3.5 across all sentiment categories. The language model’s overall average accuracy was 0.817 (SE 0.017) for long-form messages and 0.770 (SE 0.019) for short-form messages. Although accuracy varied across categories, the gap between the accuracy in ANTI and PRO sentiment classification decreased compared with GPT-3.5. For human-labeled ANTI messages, *K_20_* was 0.861 (SE 0.024) for long-form messages and 0.79 (SE 0.028) for short-form messages. For human-labeled PRO messages, *K_20_* was 0.84 (SE 0.025) for long-form messages and 0.783 (SE 0.029) for short-form messages. The accuracy for human-labeled NEU message categorization also increased compared to GPT-3.5. For NEU messages, *K_20_* was 0.685 (SE 0.044) for long-form messages and 0.703 (SE 0.045) for short-form messages. The accuracy of sentiment categories with varying *m* is reported in Table 2.

**Table 2:** Accuracy varying the number of response instances (GPT-4 Turbo).

Platform (format) and *m*^a^		ANTI^b^ (n=200)		PRO^c^ (n=200)		NEU^d^ (n=100)		All (N=500)	
		*K*_*m*_^e^ (SE)	*K*_*m*_/*K*_*20*_, %	*K*_*m*_ (SE)	*K*_*m*_/*K*_*20*_, %	*K*_*m*_ (SE)	*K*_*m*_/*K*_*20*_, %	*K*_*m*_ (SE)	*K*_*m*_/*K*_*20*_, %
**Facebook (Long format)**									
	1	0.856 (0.023)	99.4	0.828 (0.024)	98.5	0.661 (0.038)	96.5	0.806 (0.016)	98.6
	3	0.859 (0.024)	99.7	0.834 (0.025)	99.3	0.681 (0.041)	99.3	0.813 (0.016)	99.5
	5	0.860 (0.024)	99.9	0.837 (0.025)	99.6	0.685 (0.042)	100	0.816 (0.016)	99.8
	7	0.860 (0.024)	99.9	0.837 (0.025)	99.6	0.687 (0.042)	100	0.816 (0.017)	99.8
	9	0.860 (0.024)	99.9	0.838 (0.025)	99.7	0.687 (0.043)	100	0.816 (0.017)	99.9
	11	0.860 (0.024)	99.9	0.838 (0.025)	99.8	0.688 (0.043)	100	0.817 (0.017)	100
	20	0.861 (0.024)	—^f^	0.840 (0.025)	—	0.685 (0.044)	—	0.817 (0.017)	—
**Twitter (Short format)**									
	1	0.789 (0.027)	99.9	0.773 (0.028)	98.7	0.704 (0.040)	100	0.765 (0.018)	99.4
	3	0.789 (0.028)	99.9	0.776 (0.028)	99.2	0.708 (0.042)	100	0.768 (0.018)	99.8
	5	0.789 (0.028)	99.9	0.778 (0.028)	99.4	0.707 (0.043)	100	0.768 (0.018)	99.8
	7	0.790 (0.028)	100	0.779 (0.028)	99.6	0.706 (0.043)	100	0.769 (0.018)	99.9
	9	0.789 (0.028)	99.9	0.780 (0.029)	99.7	0.705 (0.044)	100	0.769 (0.018)	99.9
	11	0.789 (0.028)	99.9	0.781 (0.029)	99.7	0.705 (0.044)	100	0.769 (0.018)	99.9
	20	0.790 (0.028)	—	0.783 (0.029)	—	0.703 (0.045)	—	0.770 (0.019)	—

^a^*m* indicates the number of response instances used for majority determination. When *m* is greater than 1, machine decision is the majority among the response instances. When *m* equals 1, the machine decision is equal to the response instance (1-shot determination).

^b^anti-HTP messages.

^c^pro-HTP messages.

^d^neutral messages.

^e^*K_m_* indicates the average of the machine accuracy of *n* messages when the machine decision for each message was determined based on *m* response instances.

^f^Not applicable.

GPT-4 Turbo showed fewer false selections of the IR label across all sentiment categories. When examined with 3 randomly selected response instances (*m*=3), the model misclassified only 10 out of the entire 1000 sample messages as IR. This impacted the pattern of mismatches between human and language model sentiment classification. For instance, the model categorized 14% (28/200) of human-labeled ANTI messages as one of the other labels. Of these mismatches, 93% (26/28) were classified as NEU. Multimedia Appendix 1 includes contingency tables (Tables S3 and S8-S11) as well as flow charts (Figure S2) providing more detailed comparative descriptions of human and language model sentiment labeling.

For long-form messages, the model’s accuracy of ANTI classification based on 20 response instances was significantly higher than NEU classification (*U*=12,561.5; *P*<.001) but not significantly different from the accuracy of PRO classification (*U*=20,810.5; *P*=.30). PRO classification also showed significantly higher accuracy compared with NEU classification (*U*=12,176; *P*<.001). This pattern was observed across all *m* values. For short-form messages with lower m values, the accuracy of ANTI and PRO classification was significantly greater than NEU labeling. For example, the 1-shot determination (*K_1_*) for ANTI and PRO classification was significantly higher than for NEU classification (ANTI vs NEU: *U*=11,829; *P*<.001 and PRO vs NEU: *U*=11,625; *P*<.001). However, these differences diminished as *m* increased; *K_20_* was not significantly different across the 3 sentiment categories. The difference test results across all *m* values are provided in Multimedia Appendix 1 (Table S14 and S15).

**Figure 2:**
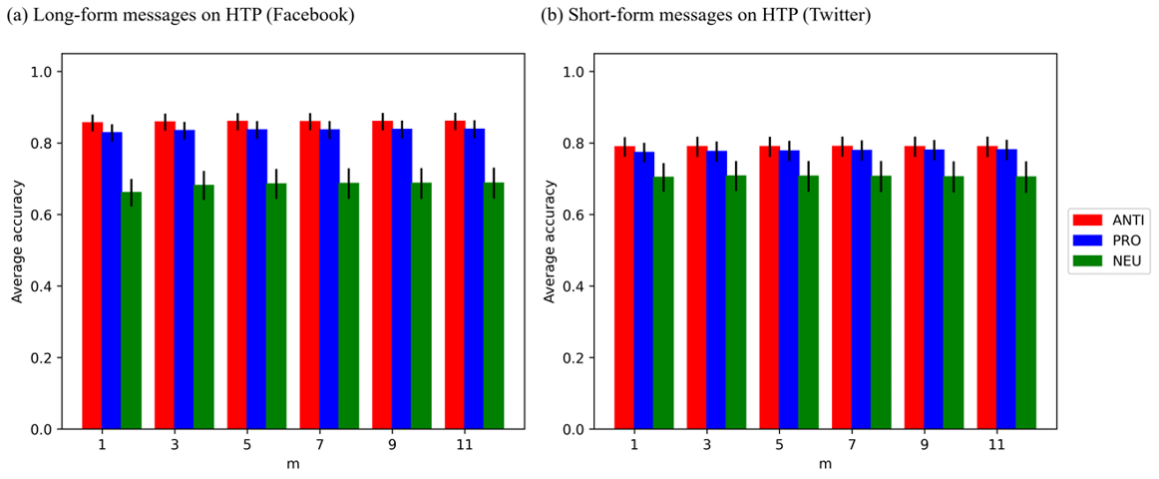
Accuracy across response instances and message formats (GPT-4 Turbo). HTP: heated tobacco product; ANTI: anti-HTP; PRO: pro-HTP; NEU: neutral.

The model’s accuracy in 1-shot cases was already comparable with that of 20 instances, as shown in Figure 2. Error bars represent the mean (SE) of the mean, and “m” refers to the number of response instances. Table 2 above presented that the accuracy of the 1-shot determination (*K_1_*) reached at least 96.5% accuracy based on 20 response instances (*K_20_*).

## Discussion

GPT-4 Turbo accurately replicated 81.7% of human sentiment evaluations for long-form messages and 77% for short-form messages, based on 20 AI responses. In comparison, GPT-3.5’s *K_20_* indicated that the model’s labeling decisions matched human coders’ evaluations with 61.2% accuracy for long-form messages and 57% accuracy for short-form messages. In sum, GPT-4 Turbo showed improvements in accuracy compared to GPT-3.5 due to increased accuracy across all sentiment categories.

Focusing on GPT-4 Turbo, which showed better overall accuracy, the LLM demonstrated already high accuracy with a small number of responses. The difference in accuracy between a small number of responses (eg, *m*=1, 3) and a high number of responses (eg, *m*=20) was not statistically significant. For all sentiment categories, the language model’s *K_3_* reached at least 99.2% of *K_20_*. The model demonstrated similar levels of accuracy for ANTI and PRO labels for both long-form and short-form messages. While the accuracy of NEU classification was lower than that of ANTI and PRO classification, it increased by approximately 20 percentage points compared with GPT-3.5. These findings suggest that GPT-4 Turbo can yield more accurate sentiment classification decisions, even with a small number of response instances, such as 3.

GPT-3.5 showed discrepancies in accuracy across sentiment categories. Specifically, the accuracy of the ANTI classification was better than the PRO classification. This finding suggests the possibility of a relative underrepresentation of messages with positive sentiment compared with negative sentiment when using LLMs for sentiment analysis of tobacco-related social media discourses. This issue calls for further exploration of approaches, techniques, and procedures to assess, mitigate, or compensate for LLMs’ potential inconsistencies across different sentiment categories, as well as the reasons underlying these discrepancies.

Employing a newer model may be the most straightforward solution, as shown by the comparison between older (GPT-3.5) and newer (GPT-4 Turbo) models. The newer model not only improved accuracy across all sentiment categories but also showed no significant difference in accuracy between ANTI and PRO classifications. This is particularly important for tobacco prevention researchers, as the detection of ANTI and PRO sentiments is important due to their possible associations with tobacco prevention outcomes [11,12,31]. However, a more recent model may not always perform better than its predecessors. For example, GPT-4 Turbo experienced a “laziness” issue, where the model does not complete user requests [32]. Therefore, the performance of new language models on specific tasks should also be rigorously tested.

Using language models specialized for health and medical information analyses, such as Google’s Med-PaLM [33] and Stanford University’s BioMedLM (previously PubMedGPT) [34], may influence accuracy. Research in this area is still emerging, with limited evidence on the application of these specialized LLMs for sentiment analysis. Previous studies have primarily used standard GPT models [35-38]. In addition, using these specialized models could be more difficult than widely used platforms like ChatGPT. Still, their performance in analyzing the sentiment of public health-related social media messages warrants future investigations, considering their capabilities in handling content from the general public, not just academic researchers and professionals.

Prompt engineering could be another strategy for improving the accuracy of LLMs in sentiment analysis and reducing discrepancies across categories. In line with the objective of this study to facilitate a straightforward comparison between human coders’ and language models’ labeling decisions, we used a prompt that closely mirrored the coding scheme for human evaluators. However, different prompting techniques can lead to different results for similar requests [39,40]. Techniques such as few-shot prompting, which involves including task-related examples within the prompts, may enhance accuracy. For instance, rather than only defining sentiment labels, the coding scheme can provide several example messages for each label. Although these techniques complicate direct comparisons between human and machine classification, they possibly offer potential accuracy gains worth exploring.

Establishing and adopting coding procedures for LLM-involved coding is also worthwhile. A study investigating LLMs as substitutes for human coders in labeling texts on political topics serves as a good example [30]. The authors proposed a “hybrid” model where disagreements between the “GPT-4 first run” and the “GPT-4 second run” are resolved by a human coder. Their findings demonstrated that the hybrid approach can be optimized with minimal additional human effort and boosted the accuracy of GPT-4’s annotations. This hybrid approach can potentially be adapted for the analysis of sentiment about health topics, and other coding procedures should be explored to further enhance the accuracy and efficiency.

The implications and future applications of our findings should be discussed with caution. First, this study is a focused case study on OpenAI’s GPT, examining sentiment analysis on Twitter and Facebook messages related to HTPs. Future research can extend beyond this specific focus to evaluate the accuracy of LLMs in analyzing the sentiment of health-related information across a broader range of topics and platforms. Second, while the differences in accuracy for ANTI and PRO sentiment classifications that were present in GPT-3.5 disappeared in GPT-4 Turbo, and the accuracy of NEU classification increased by 20%, the NEU classification accuracy remained lower. A simple explanation might be the underperformance of the models. For instance, GPT-4 Turbo misclassified a human-labeled NEU message, which used HTPs as examples to explain an economic principle, as irrelevant. Alternatively, this difference might stem from the inherent complexity of evaluating neutrality [15,41]. For example, human coders classified a message as NEU, describing IQOS (Philip Morris International) as a device that uses a “patented heat-control technology.” In contrast, GPT-4 Turbo classified the same message as PRO, interpreting “patented heat-control technology” to have positive connotations. It may be worthwhile for future research to explore the patterns of misclassifications by LLMs. Third, this study did not address the potential ethical issues of using LLMs for sentiment analysis of social media content. Ethical considerations, such as security, privacy protection, and data ownership, are important when using LLMs to analyze social media messages [42,43]. These issues require careful attention when analyzing health-related information using LLMs, too [44,45]. Future research should use LLMs while carefully considering the potential ethical issues surrounding their content analyses.

## Appendix

Multimedia Appendix 1 Online supplements (Table S1-S16, Figure S1 and Figure S2).

## Abbreviations

AI: artificial intelligence
CT: CrowdTangle
HTP: heated tobacco product
LLM: large language model

## Data Availability

The datasets generated during and/or analyzed during this study are available in the Open Science Framework (OSF) repository [46].
