# Supplementary material for: Large Language Models’ Accuracy in Emulating Human Experts’ Evaluation of Public Sentiments about Heated Tobacco Products on Social Media: Evaluation Study
Source: J Med Internet Res. 2025 Mar 4;27:e63631. doi: 10.2196/63631 (PMC11920658; doi:10.2196/63631)
Supplement: Multimedia Appendix 1 [file jmir_v27i1e63631_app1.pdf]

Online Supplements for "Large Language Models' Accuracy in Emulating Human Experts' Evaluation of Public Sentiments about Heated Tobacco Products on Social Media: Evaluation Study"

Kwanho Kim, PhD[1] & Soojong Kim, PhD[2]

[1] Department of Media, College of Politics and Economics, Kyung Hee University, Korea

[2] Department of Communication, University of California Davis, United States

Author Note

Soojong Kim https://orcid.org/0000-0002-1334-5310

Correspondence concerning this article should be addressed to Soojong Kim, 1 Shields Ave, Kerr Hall #361, Davis, CA 95616, United States. Email: sjokim@ucdavis.edu

Human Evaluation Dataset Generation Process

The human-annotated datasets were generated in the following four steps. The multi-step random sampling procedure was designed to enhance the likelihood of including messages with potentially positive and negative sentiments, ensuring their inclusion in the selected messages for the human annotation. For short-form messages, first, from the entire message pool of 60,031 tweets, 200 messages were randomly sampled. Two human evaluators independently classified these messages into one of the following five categories: ANTI (anti-HTPs messages), PRO (pro-HTPs messages), NEU (neutral messages), MIX (messages containing a mixture of positive and negative attitudes on HTPs), and IR (messages not relevant to HTPs). After completing categorizations, evaluators checked differences in categorization decisions between evaluators and resolved these discrepancies via discussion. Second, using these 200 human-evaluated messages, we developed preliminary machine-learning ANTI and PRO binary classifiers to analyze the sentiment of all messages in the entire dataset. These classifiers were developed using DistilBERT, a widely-used pre-trained machine-learning model [1]. Third, using these preliminary machine-classification results, we randomly sampled an additional 500 ANTI and 500 PRO messages from the pool. Lastly, a team of three evaluators analyzed the sentiment of these 1,000 messages. Messages were first evaluated independently by two evaluators. The role of a third evaluator was to resolve disagreements in sentiment classifications between these two initial evaluators. Each initial evaluator was either a postdoctoral-level public health communication scientist or an undergraduate student who had received specialized training and exercise for this sentiment analysis task. The third evaluator was a postdoctoral-level scholar specializing in social media and health communication research. The inter-coder reliability of the evaluators, measured with Cohen’s Kappa, was .885.

For long-form samples, 1,250 samples were prepared using a similar procedure with a few additional steps. The first and second steps were identical to those for short-form samples. 200 messages were randomly sampled from the entire message pool of 16,284 Facebook posts and then analyzed for sentiment by two independent human evaluators to prepare training materials for preliminary classifiers. However, the preliminary classification results showed that there were only 144 PRO messages in the entire long-form message pool. Prior to the next step, preliminary classifications were re-conducted using improved preliminary classifiers re-trained with 250 samples. These 250 samples were prepared by adding 50 more human-evaluated random samples to the 200 samples that had already been evaluated by human evaluators for sentiment. Using these re-trained preliminary classifiers, the sentiment of all long-form messages was analyzed. Identical to the short-form message sampling procedure, 500 ANTI and 500 PRO messages were randomly sampled from the entire dataset using preliminary classification results from the re-trained classifiers. Then, these 1,000 messages were evaluated by three human evaluators, as they did for short-form samples. The reliability measure, represented by Cohen's Kappa, was .938.

References

1. Sanh V, Debut L, Chaumond J, Wolf T. DistilBERT, a distilled version of BERT: smaller, faster, cheaper and lighter. arXiv preprint arXiv:191001108 2019;

Table S1. Prompt for Machine Evaluation

Prompt
The content presented in [Message] is a post on {Platform}. [Message] contains content generated or re-shared by an author. What does the author of the post argue? Classify the post into one of the categories explained in [Scheme]. Start your response with a category code you choose (IR, PRO, ANTI, MIX, NEU) without any preceding characters or information before it. Then, print a colon ': ' following the code, and proceed to explain your decision.<br><br>[Message]<br>{Content}<br><br>[Scheme]<br>Below, HTP stands for heated tobacco products, also known as HNB (heat-not-burn) tobacco products or tobacco heating products. HTP brands include IQOS, Glo, Eclipse, and Ploom.<br>1.Irrelevant messages (IR): Use this label if a message is not related to HTP.<br>2.Pro-HTP messages (PRO): Use this label if a message is primarily about HTP and clearly supportive of the use of HTP, the HTP industry, and policies allowing and promoting the use of HTP.<br>3.Anti-HTP messages (ANTI): Use this label if a message is primarily about HTP and clearly against the use of HTP, the HTP industry, and policies preventing and discouraging the use of HTP.<br>4.Mixed messages (MIX): Use this label if a message is primarily about HTP and contains balanced information that both supports and opposes HTP.<br>5.Neutral or 'Not Applicable' messages (NEU): Use this label if a message meets one or more of the following criteria: (a) the message is not primarily about HTP, (b) the length of the message is too short to understand its meaning, or (c) the message neither supports nor opposes the use of HTP, the HTP industry, and policies influencing the use of HTP.

Example response instances
• "ANTI: The author of the post is arguing against the use of heated tobacco products (HTP) by stating that they contain nicotine and emphasizing the need for more research to understand the health effects of these products."<br>• "PRO: The author of the post argues in favor of the proposed sin tax reform, which aims to raise the excise tax rate on alcohol, heated tobacco products (HTPs), and vaping products."

Note. The placeholder {Platform} was substituted with either "Facebook" or "Twitter", while {Content} was substituted with the actual content of a social media post.

Comparative Cross-Tabulation of Human and GPT Sentiment Evaluations

Due to the probabilistic nature of LLMs, a message's category determined by GPT models can vary across different response instances. Considering these variations, Table S2 and S3 were calculated based on the following procedure. First, for each message, three out of 20 response instances were randomly selected with replacement. Second, the majority sentiment among the three selected instances was determined. Third, the first and second processes were conducted for all messages, and a cross-tabulation result was calculated by comparing the human- and machine-evaluations of the messages. We repeated these processes 1,000 times, and then the 1,000 cross-tabulation results generated from these iterations were averaged.

Table S2. Comparative Cross-Tabulation of Human and GPT-3.5 Sentiment Evaluations

Facebook (Long format)				
GPT-3.5	Human			
	ANTI	PRO	NEU	Total
ANTI	151.123 (.084)<br>75.6%	13.691 (.055)<br>6.8%	8.987 (.051)<br>9.0%	173.801<br>34.8%
PRO	5.788 (.037)<br>2.9%	109.005 (.085)<br>54.5%	8.641 (.044)<br>8.6%	123.434<br>24.7%
NEU	30.139 (.081)<br>15.1%	29.771 (.093)<br>14.9%	46.060 (.093)<br>46.1%	105.970<br>21.2%
MIX	0.020 (.005)<br>0.01%	0.529 (.021)<br>0.2%	0.465 (.014)<br>0.5%	1.014<br>0.2%
IR	12.930 (.075)<br>6.5%	47.068 (.099)<br>23.5%	35.783 (.089)<br>35.8%	95.781<br>19.1%
Total	200<br>100%	200<br>100%	100<br>100%	500<br>100%
Twitter (Short format)				
GPT-3.5	Human			
	ANTI	PRO	NEU	Total
ANTI	139.130 (.083)<br>69.6%	27.454 (.064)<br>13.7%	12.754 (.051)<br>12.8%	179.338<br>35.9%
PRO	4.757 (.045)<br>2.4%	95.225 (.081)<br>47.6%	9.278 (.053)<br>9.3%	109.260<br>21.9%
NEU	40.220 (.092)<br>20.1%	48.818 (.101)<br>24.4%	50.647 (.085)<br>50.6%	139.685<br>27.9%
MIX	0.411 (.017)<br>0.2%	0.842 (.027)<br>0.4%	1.882 (.032)<br>1.9%	3.135<br>0.6%
IR	15.482 (.080)<br>7.7%	27.661 (.096)<br>13.8%	25.439 (.081)<br>25.4%	68.582<br>13.7%
Total	200 (100%)	200 (100%)	100 (100%)	500 (100%)

Note. The table includes counts and standard errors (noted in parentheses), along with column proportions.

Table S3. Comparative Cross-Tabulation of Human and GPT-4 Turbo Sentiment Evaluations

Facebook (Long format)				
GPT-4 Turbo	Human			
	ANTI	PRO	NEU	Total
ANTI	171.708 (.040)<br>85.9%	3.029 (.008)<br>1.5%	11.321 (.024)<br>11.3%	186.058<br>37.2%
PRO	0.233 (.014)<br>0.1%	166.906 (.056)<br>83.5%	15.148 (.054)<br>15.1%	182.287<br>36.5%
NEU	25.963 (.039)<br>13.0%	26.401 (.058)<br>13.2%	68.097 (.040)<br>68.1%	120.461<br>24.1%
MIX	2.071 (.026)<br>1.0%	2.354 (.017)<br>1.2%	1.694 (.029)<br>1.7%	6.119<br>1.2%
IR	0.025 (.005)<br>0.0%	1.31 (.031)<br>0.7%	3.74 (.044)<br>3.7%	5.075<br>1.0%
Total	200<br>100%	200<br>100%	100<br>100%	500<br>100%
Twitter (Short format)				
GPT-4 Turbo	Human			
	ANTI	PRO	NEU	Total
ANTI	157.789 (.047)<br>78.9%	5.311 (.021)<br>2.7%	8.69 (.030)<br>8.7%	171.79<br>34.4%
PRO	4.233 (.020)<br>2.1%	155.363 (.058)<br>77.7%	15.569 (.035)<br>15.6%	175.165<br>35.0%
NEU	32.963 (.054)<br>16.5%	32.031 (.052)<br>16.0%	70.721 (.059)<br>70.7%	135.715<br>27.1%
MIX	4.383 (.027)<br>2.2%	7.148 (.033)<br>3.6%	1.166 (.021)<br>1.2%	12.697<br>2.5%
IR	0.632 (.020)<br>0.3%	0.147 (.011)<br>0.1%	3.854 (.028)<br>3.9%	4.633<br>0.9%
Total	200 (100%)	200 (100%)	100 (100%)	500 (100%)

Note. The table includes counts and standard errors (noted in parentheses), along with column proportions.

Binary Confusion Matrix of Human and GPT Models' Sentiment Evaluations

In this study, we transformed the multi-category sentiment classifications made by human evaluators and GPT models into binary classifications to generate confusion matrices for ANTI and PRO classifications. For the ANTI classifications, we grouped PRO, NEU, MIX, and IR as 'Non-ANTI,' to generate binary confusion matrices. Similarly, for the PRO classifications, we grouped ANTI, NEU, MIX, and IR as 'Non-PRO,' to create binary confusion matrices. The data used for these comparisons were derived from the cross-tabulation analyses results reported in Table S2 and S3.

Table S4. Binary Confusion Matrix of ANTI Classification of Facebook Messages (GPT-3.5)

		**Human Coders' Evaluation**	
		ANTI	Non-ANTI
**GPT-3.5's Evaluation**	ANTI	151.123	22.678
	Non-ANTI	48.877	277.322

Note.
True Positive Rate (Sensitivity) = .756
False Positive Rate (False Alarm) = .076
True Negative Rate (Specificity) = .924
False Negative Rate (Miss) = .244

Precision = .869
Recall = .756
F1 = .808

Table S5. Binary Confusion Matrix of PRO Classification of Facebook Messages (GPT-3.5)

		**Human Coders' Evaluation**	
		PRO	Non-PRO
**GPT-3.5's Evaluation**	PRO	109.005	14.429
	Non-PRO	90.995	285.571

Note.
True Positive Rate (Sensitivity) = .545
False Positive Rate (False Alarm) = .048
True Negative Rate (Specificity) = .952
False Negative Rate (Miss) = .455

Precision = .883
Recall = .545
F1 = .674

Table S6. Binary Confusion Matrix of ANTI Classification of Twitter Messages (GPT-3.5)

		**Human Coders' Evaluation**	
		ANTI	Non-ANTI
**GPT-3.5's Evaluation**	ANTI	139.13	40.208
	Non-ANTI	60.87	259.792

Note.
True Positive Rate (Sensitivity) = .696
False Positive Rate (False Alarm) = .134
True Negative Rate (Specificity) = .866
False Negative Rate (Miss) = .304

Precision = .776
Recall = .696
F1 = .734

Table S7. Binary Confusion Matrix of PRO Classification of Twitter Messages (GPT-3.5)

		**Human Coders' Evaluation**	
		PRO	Non-PRO
**GPT-3.5's Evaluation**	PRO	95.225	14.035
	Non-PRO	104.775	285.965

Note.
True Positive Rate (Sensitivity) = .476
False Positive Rate (False Alarm) = .047
True Negative Rate (Specificity) = .953
False Negative Rate (Miss) = .524

Precision = .872
Recall = .476
F1 = .617

Table S8. Binary Confusion Matrix of ANTI Classification of Facebook Messages (GPT-4 Turbo)

		**Human Coders' Evaluation**	
		ANTI	Non-ANTI
**GPT-4 Turbo's Evaluation**	ANTI	171.708	14.35
	Non-ANTI	28.292	285.65

Note.
True Positive Rate (Sensitivity) = .858
False Positive Rate (False Alarm) = .048
True Negative Rate (Specificity) = .952
False Negative Rate (Miss) = .142

Precision = .923
Recall = .858
F1 = .889

Table S9. Binary Confusion Matrix of PRO Classification of Facebook Messages (GPT-4 Turbo)

		**Human Coders' Evaluation**	
		PRO	Non-PRO
**GPT-4 Turbo's Evaluation**	PRO	166.906	15.381
	Non-PRO	33.094	284.619

Note.
True Positive Rate (Sensitivity) = .834
False Positive Rate (False Alarm) = .051
True Negative Rate (Specificity) = .949
False Negative Rate (Miss) = .166

Precision = .916
Recall = .834
F1 = .873

Table S10. Binary Confusion Matrix of ANTI Classification of Twitter Messages (GPT-4 Turbo)

		Human Coders' Evaluation	
		ANTI	Non-ANTI
**GPT-4 Turbo's Evaluation**	ANTI	157.789	14.048
	Non-ANTI	42.211	286.119

Note.
True Positive Rate (Sensitivity) = .789
False Positive Rate (False Alarm) = .047
True Negative Rate (Specificity) = .953
False Negative Rate (Miss) = .211

Precision = .918
Recall = .789
F1 = .849

Table S11. Binary Confusion Matrix of PRO Classification of Twitter Messages (GPT-4 Turbo)

		Human Coders' Evaluation	
		PRO	Non-PRO
**GPT-4 Turbo's Evaluation**	PRO	155.363	19.802
	Non-PRO	44.637	280.198

Note.
True Positive Rate (Sensitivity) = .777
False Positive Rate (False Alarm) = .066
True Negative Rate (Specificity) = .934
False Negative Rate (Miss) = .223

Precision = .887
Recall = .777
F1 = .828

Figure S1. Comparative Visualizations of Sentiment Evaluations by Humans and GPT-3.5 on the Same Messages

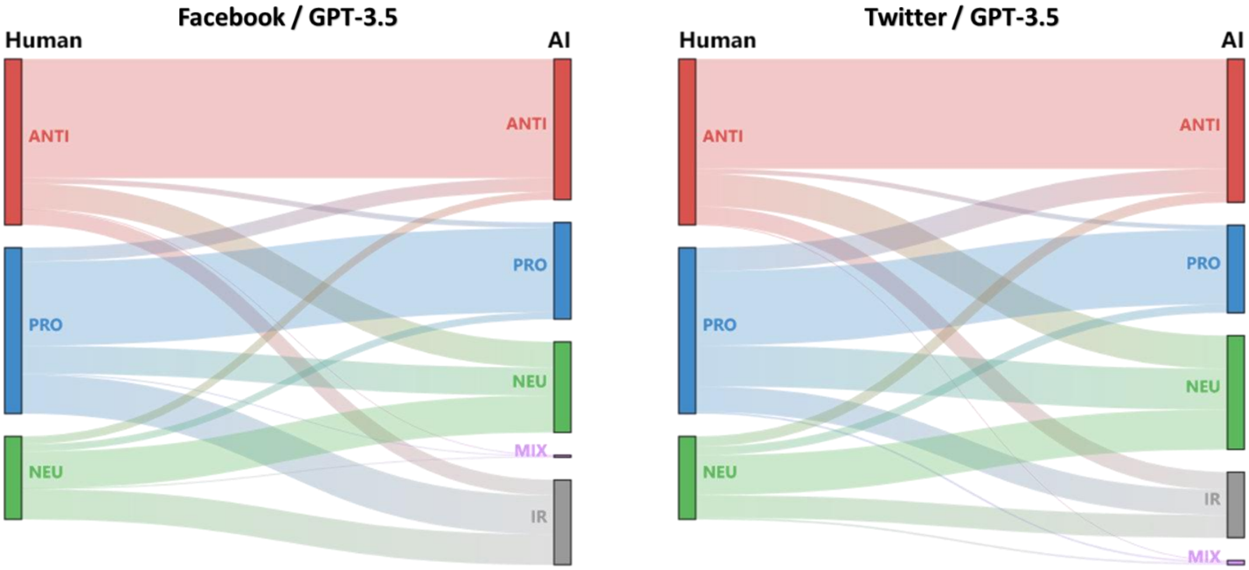

Note. The diagrams provide comparative illustrations of the sentiment labeling decisions on the same set of Facebook and Twitter messages, made by human evaluators and GPT-3.5.

Figure S2. Comparative Visualizations of Sentiment Evaluations by Humans and GPT-4 Turbo on the Same Messages

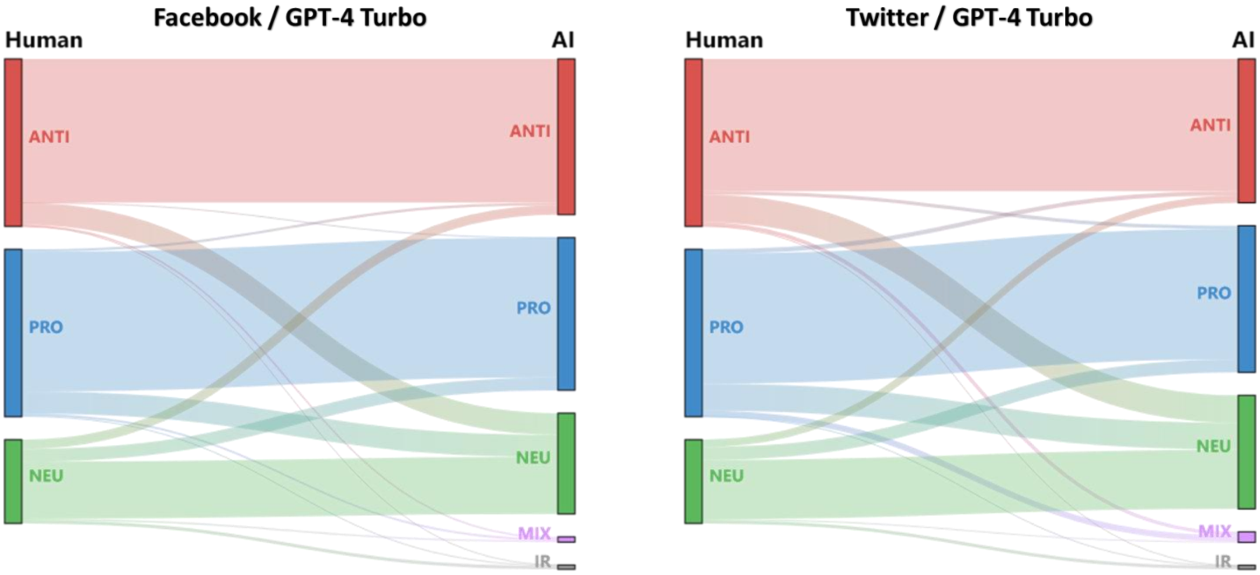

Note. The diagrams provide comparative illustrations of the sentiment labeling decisions on the same set of Facebook and Twitter messages, made by human evaluators and GPT-4 Turbo.

Table S12. Difference in Average Accuracy Between Evaluation Sets (Facebook / GPT-3.5)

m	**Eval Set 1**	**Eval Set 2**	**Mann-Whitney *U***	***p***
1	ANTI	PRO	26988.5	<.001
1	ANTI	NEU	14390.5	<.001
1	PRO	NEU	10240	.74
3	ANTI	PRO	26784.5	<.001
3	ANTI	NEU	14697	<.001
3	PRO	NEU	10709	.32
5	ANTI	PRO	26587	<.001
5	ANTI	NEU	14837.5	<.001
5	PRO	NEU	10857	.23
7	ANTI	PRO	26697	<.001
7	ANTI	NEU	14854.5	<.001
7	PRO	NEU	10930.5	.19
9	ANTI	PRO	26594.5	<.001
9	ANTI	NEU	14823	<.001
9	PRO	NEU	10945.5	.18
11	ANTI	PRO	26357	<.001
11	ANTI	NEU	14821	<.001
11	PRO	NEU	11029.5	.14
20	ANTI	PRO	26483.5	<.001
20	ANTI	NEU	14675.5	<.001
20	PRO	NEU	10987.5	.16

Table S13. Difference in Average Accuracy Between Evaluation Sets (Twitter / GPT-3.5)

m	**Eval Set 1**	**Eval Set 2**	**Mann-Whitney *U***	***p***
1	ANTI	PRO	26866	<.001
1	ANTI	NEU	13192.5	<.001
1	PRO	NEU	9088.5	.20
3	ANTI	PRO	26827.5	<.001
3	ANTI	NEU	13425	<.001
3	PRO	NEU	9357	.36
5	ANTI	PRO	26760	<.001
5	ANTI	NEU	13423	<.001
5	PRO	NEU	9345	.36
7	ANTI	PRO	26444	<.001
7	ANTI	NEU	13402	<.001
7	PRO	NEU	9440	.43
9	ANTI	PRO	26466.5	<.001
9	ANTI	NEU	13322	<.001
9	PRO	NEU	9413.5	.41
11	ANTI	PRO	26243.5	<.001
11	ANTI	NEU	13374.5	<.001
11	PRO	NEU	9500	.48
20	ANTI	PRO	25876.5	<.001
20	ANTI	NEU	13312.5	<.001
20	PRO	NEU	9603.5	.57

Table S14. Difference in Average Accuracy Between Evaluation Sets (Facebook / GPT-4 Turbo)

m	**Eval Set 1**	**Eval Set 2**	**Mann-Whitney *U***	***p***
1	ANTI	PRO	21271.5	.15
1	ANTI	NEU	14308	< .001
1	PRO	NEU	13674	< .001
3	ANTI	PRO	21270	.15
3	ANTI	NEU	14281.5	< .001
3	PRO	NEU	13654.5	< .001
5	ANTI	PRO	21338	.12
5	ANTI	NEU	14190	< .001
5	PRO	NEU	13549.5	< .001
7	ANTI	PRO	21186.5	.16
7	ANTI	NEU	13506.5	< .001
7	PRO	NEU	12938.5	< .001
9	ANTI	PRO	20926	.26
9	ANTI	NEU	13269.5	< .001
9	PRO	NEU	12796	< .001
11	ANTI	PRO	20926.5	.25
11	ANTI	NEU	13158.5	< .001
11	PRO	NEU	12692.5	< .001
20	ANTI	PRO	20810.5	.30
20	ANTI	NEU	12561.5	< .001
20	PRO	NEU	12176	< .001

Table S15. Difference in Average Accuracy Between Evaluation Sets (Twitter / GPT-4 Turbo)

m	**Eval Set 1**	**Eval Set 2**	**Mann-Whitney *U***	***p***
1	ANTI	PRO	20222	.81
1	ANTI	NEU	11829	.003
1	PRO	NEU	11625	.008
3	ANTI	PRO	20218	.82
3	ANTI	NEU	11812	.003
3	PRO	NEU	11626	.008
5	ANTI	PRO	20152	.87
5	ANTI	NEU	11596	.008
5	PRO	NEU	11431.5	.02
7	ANTI	PRO	20496	.56
7	ANTI	NEU	11494.5	.011
7	PRO	NEU	11212.5	.04
9	ANTI	PRO	20673.5	.45
9	ANTI	NEU	11562.5	.007
9	PRO	NEU	11223	.04
11	ANTI	PRO	20828.5	.35
11	ANTI	NEU	11235.5	.03
11	PRO	NEU	10837	.15
20	ANTI	PRO	20385	.66
20	ANTI	NEU	10932	.09
20	PRO	NEU	10744.5	.18

Table S16. Human and GPT-4 Turbo (GPT-4T) Evaluation Examples

Facebook Messages	Human	GPT-4T
Big Tobacco's new cigarette is sleek, smokeless — but is it any better for you? Experts doubt motive, science behind new heated tobacco tech. FDA will decide soon whether to allow its sale. Tobacco companies have a long history of lying about their deadly products, so any claims about safer tobacco products must be regarded with skepticism. To protect public health, we need strong FDA regulation of all tobacco products and any health claims about them.	ANTI	ANTI
The IQOS system is an elegant and innovative alternative to smoking using tobacco instead of Eliquids. IQOS doesn't burn the tobacco it heats it for the nicotine and real tobacco taste, If you'd like to learn more and have a look in person, just pop into one of our stores and our expert staff will do their utmost to help you quit the ciggies for good! ================================ #photooftheday #photography #vapenation #vapeporn #vape #eliquid #eliquids #vaping #vapers #vapelyfe #vapefam #vapor #vapelife #ejuice #vaper #vapecommunity #vapedaily #instavape #ejuices #vapeon #vapestagram #vapepics #vapetricks #vapelove #cloudchaser #vapeshop #vapejuice #subohm	PRO	PRO
Heated tobacco products (HTPs) like IQOS and Eclipse, sometimes marketed as "heat-not-burn" technology, represent a diverse class of products that heat the tobacco leaf to produce an inhaled aerosol. They are different from e-cigarettes, which heat a liquid that can contain nicotine derived from tobacco.	NEU	NEU
Tobacco giant grapples with rules for marketing vaping product The authorities hold firm to the view that a hybrid tobacco product is still tobacco – and harmful – even if less harmful than smoking traditional cigarettes A store without a name -- and there are others. The no-name IQOS store sells vaping items but the catch here is that the vaping is done with tobacco rather than a liquid with (often) nicotine in it that is heated and turns to steam (rather than burns). Confused about the different products and tobacco companies' push for change? I tried to help walk readers through this confusing maze. Read my story.	ANTI	NEU
It is important that we continue to modernize proven tobacco prevention and control strategies to include newer products entering the market such as Heated Tobacco Products.	PRO	NEU
Making bioeconomy circular: How far can circular economy principles be applied to the bioeconomy? The European Commission has adopted an ambitious new Circular Economy Package to help European businesses and consumers make the transition to a stronger and more circular economy where resources are… Electronic cigarettes and heated tobacco products are a rapidly evolving category. Industry claims there is a need to regulate but in a proportionate manner.	NEU	IR

Twitter Messages	Human	GPT-4T
109 (2-24): New heated #tobacco device causes same damage to #lung cells as #e_cigs and #smoking	ANTI	ANTI
IQOS is billed as the most successful smoke-free product, with more than 12 million users around the world. Around 9 million IQOS users have completely stopped smoking.	PRO	PRO
'Heat-not-burn' cigarettes on their way to U.S. market	NEU	NEU
"Heat-not-burn products, unlike their e-cigarette counterparts, do contain tobacco."<br>#cigarette 0<br>#cancer 1<br>So #vape https://t.co/BPZdf4Ye0U	ANTI	NEU
Too bad that they have no Iqos in ur shitty country	PRO	NEU
IQOS devices use a patented heat-control technology that precisely heats tobacco-filled sticks wrapped in paper, without the burning, to release a water-based aerosol – not smoke.	NEU	PRO
